# Fra2, a potential therapeutic target for silicosis

**DOI:** 10.1016/j.gendis.2025.101723

**Published:** 2025-06-18

**Authors:** Yuanmeng Qi, Jiarui Xia, Xuesong Zhang, Xiaoying Li, Qimeng Li, Zhenzhen Yang, Wu Yao, Changfu Hao, Youliang Zhao

**Affiliations:** a Department of Occupational and Environment Health, College of Public Health, Zhengzhou University, Zhengzhou, Henan 450001, China; b Department of Child and Adolescence Health, College of Public Health, Zhengzhou University, Zhengzhou, Henan 450001, China

Silicosis is caused by long-term inhalation of large quantities of free silica dust and is characterized by pulmonary dysfunction, persistent inflammation of the lungs, formation of silicosis nodules and irreversible pulmonary fibrosis, leading to respiratory failure and eventual death. Pulmonary macrophages, as the first line of defense of the lungs, engulf and remove inhaled silica dust particles.^1^ Long-term exposure to silica dust can cause macrophages to be damaged, release various cytokines and chemokines, and induce inflammatory responses.^2^ Macrophages also activate fibroblasts during the inflammatory process, leading to the formation of fibrosis. They further exacerbate fibrosis in the lungs by secreting pro-fibrotic factors such as TGF-β, which promotes the accumulation of collagen and other extracellular matrix components.^3^ Fra-2 is a transcription factor (TF) in the AP-1 family that has received relatively little attention. Previous studies have confirmed that Fra-2 expression is up-regulated in IPF lung tissue, and Fra-2 transgenic mice exhibit spontaneous pulmonary fibrosis.^4^ TGF-β1 is a potent fibrogenic factor that plays important regulatory roles in fibrotic diseases, including cell proliferation, differentiation, migration, immunomodulation, and extracellular matrix (ECM) transformation, and is involved in tissue repair and fibrosis. Sashwati Roy et al found that deletion of the AP-1 binding site resulted in higher basal TGF-β reporter activity under 5% O_2_ conditions, suggesting that TGF-β expression was derepressed in the absence of AP-1 binding.^5^ These studies suggest a close interaction between Fra-2 and TGF-β1 signaling pathways. We speculate that Fra2 may promote pulmonary fibrosis in silicosis mice by controlling the release of TGF-β1 from macrophages.

In this study, we constructed silica-exposed 3-day, 14-day, and 56-day mouse models of silicosis (Fig. S1A). Hematoxylin and Eosin staining (HE, for histopathology) and Masson's Trichrome staining (Masson, for collagen visualization) staining showed that the alveoli of mice were obviously damaged on the 3rd day after silica exposure, and a large number of neutrophils as well as macrophage infiltration appeared; on the 14th day after dust staining, dust cells could be seen to gather in the respiratory bronchioles, blood vessels and bronchioles around the lung tissues to form dust cell granulomas; on the 56th day in the silica-exposed group of mice, the structure of the alveoli could be seen to be severely damaged, and silica nodules with collagen deposits appeared (Fig. 1A). Compared with the control group, the protein expression levels of the fibrosis-related markers Collagen I, *α*-SMA, and CTGF were significantly increased in lung tissues in a time-dependent manner at 14 d and 56 d after silica dust dyeing (Fig. 1B). And the hydroxyproline (HYP) content in the lung tissue of mice gradually increased with the prolongation of silica exposure time (Fig. 1C). The above results indicated that the mouse model of silicosis was successfully constructed at each dust exposure time point.

**Figure 1:**
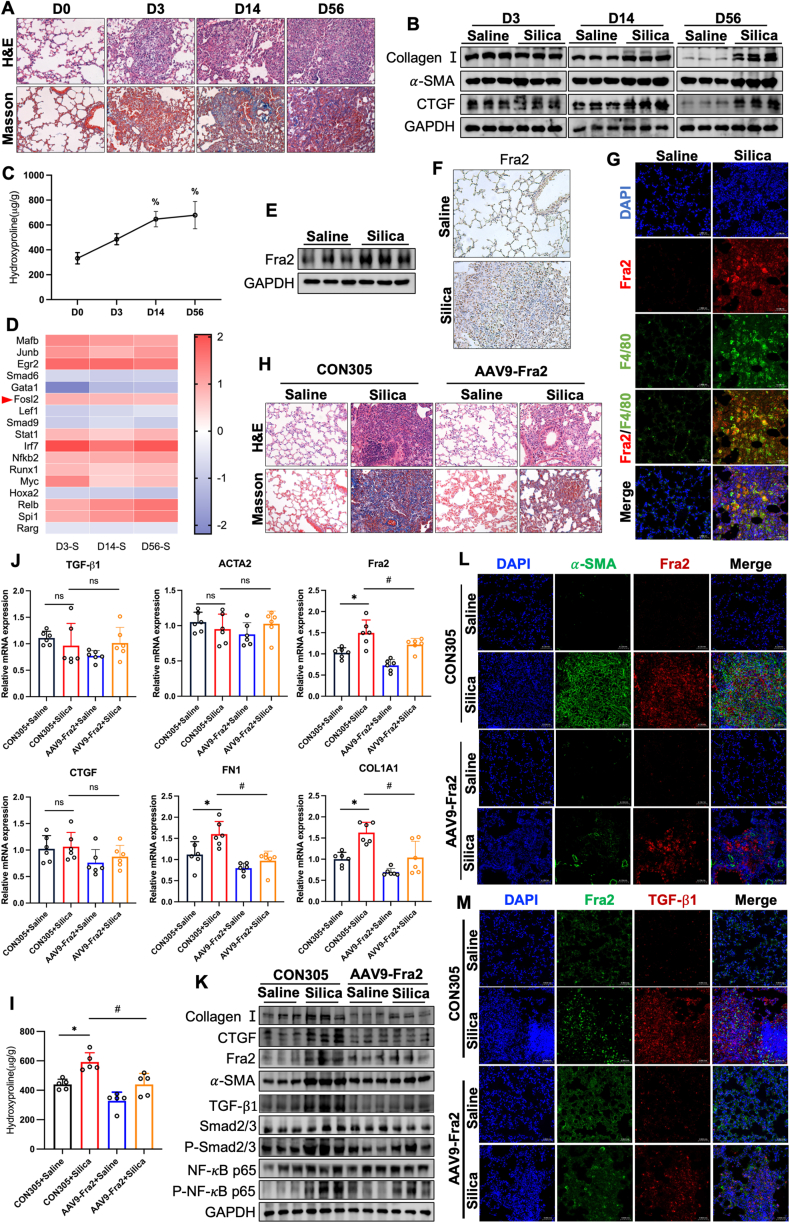
AVV9-Fra2 attenuated silica-induced pulmonary fibrosis. **(A)** HE and Masson staining showed histopathological changes and collagen deposition in mouse lungs. **(B)** Western blot detected CollagenⅠ, *α*-SMA, and CTGF expression levels in mouse lung tissues. **(C)** The HYP content was determined in mouse lung tissue after silica exposure. **(D)** Transcription factors with consistent trends across groups were displayed (blue, down-regulated; red, up-regulated). **(E)** Western blot detected Fra2 expression in mouse lung tissue after silica exposure. **(F)** IHC detected changes in Fra2 expression levels in mouse lung tissues after silica exposure. **(G)** IF observed Fra2 expression in mouse lung tissue macrophages after silica exposure. **(H)** HE and Masson staining assessed histopathologic changes and collagen deposition in mouse lungs. **(I)** RT-qPCR was used to detect the mRNA expression levels of related indicators in the lung tissue of mice. **(J)** The HYP content was determined in lung tissues of various groups of mice after exposure to silicon dioxide. **(K)** Western blot was used to detect the protein expression levels of related indicators in lung tissue of mice. **(L)** IF observed Fra2 and *α*-SMA expression in the lung tissues of mice in each group. **(M)** IF observed Fra2 and TGF-β1 expression in lung tissues of mice in each group. All data were expressed as mean ± SD. ^%^*p* < 0.05 vs. D0; ∗*p* < 0.05 vs. CON305+Saline group;^#^*p* < 0.05 vs. CON305+Silica group.

To further investigate the trend of silicosis progression, we screened four groups of mice with silicosis for time-series system-wide differential genes using RNA-seq sequencing. Principal Component Analysis (PCA) showed significant differences between groups and good biological reproducibility of samples within groups at each time point (Fig. S2A). The 3 time points were screened for a large number of differential genes (Fig. S2B). The results of differential gene clustering analysis demonstrated the expression of differential genes among different groups (Fig. S2C). Gene function analysis showed that “Cytokine−cytokine receptor interaction” and “ECM−receptor interaction” were significantly enriched (Fig. S2D). In addition, TFs annotation of the differentially expressed genes in each group revealed multiple TFs with significant differences, among which the expression level of Fra2 was enhanced as a whole (Fig. 1D). Western blot and immunohistochemistry (IHC) quantitative analysis showed that the expression level of Fra2 in lung tissue of mice exposed to silica for 56 days was significantly higher than that of the control group, and it was significantly enriched in alveolar macrophages (Fig. 1E, F). Further immunofluorescence (IF) showed that Fra2 was highly expressed in F4/80 positive macrophages (a major surface marker protein of mouse macrophages) (Fig. 1G). This suggested that Fra2-expressing macrophages may accelerate the process of pulmonary fibrosis in silicosis mice by promoting the activation of fibroblasts. However, the specific mechanism needs to be further proved.

To further demonstrate that Fra2 plays a crucial role in the development of silicosis. We silenced Fra2 expression by instillation of adeno-associated virus 9 carrying Fra2-targeting shRNA (AAV9-Fra2, 10^12^v.g/mL, 100 μL) into the lungs of mice, which were expressed for 21 days and then exposed to silica for 56 days (Fig. S3A). After 21 days of AAV9-Fra2 infusion, green fluorescent protein (GFP) expression was observed in the lungs of mice, showing high infection efficiency (Fig. S3B). HE and Masson staining showed that after AAV9-Fra2 treatment, the degree of alveolar structural disruption in the lung tissue of mice in the silica-exposed group was attenuated, with a reduction in the area of tissue fibrosis and a decrease in collagen deposition (Fig. 1H). Meanwhile AAV9-Fra2 treatment significantly suppressed the elevated Fra2 levels caused by silica exposure, the upregulation of the expression levels of the fibrogenic factor TGF-β1, the fibrosis marker FN1, Collagen Ι, CTGF, and α-SMA, as well as the inflammation-related pathways Smad2/3 and NK-κB-P65 phosphorylation levels (Fig. 1I–K). Primer sequences for all genes are shown in Supplementary Table 1. Knockdown of Fra2 significantly reduced the elevated HYP level in lung tissue of mice exposed to silica (Fig. 1J). In hypoxia-induced fibrosis, all three targets of Fra2 are directly related to TGF-β. This suggested that Fra2 may be associated with the induction of TGF-β1 transcription during the pathogenesis of silicosis in mice. In addition, IF showed that compared with the control group, the fluorescence intensity of α-SMA and TGF-β1 in the fibrotic nodules area of the lung tissue of the SiO_2_ exposure group was significantly increased, while after the intervention of AAV9-Fra2, the abnormal expression of the above molecules in the fibrotic nodules area of the lung tissue of the SiO_2_ exposure group was effectively reversed (Fig. 1L, M).

In conclusion, our findings reveal a pro-fibrotic role of the TF Fra2 in silica-exposed mice, which significantly attenuates silica exposure-induced lung fibrosis in silica-exposed mice after silencing of highly expressed Fra2 in silica-exposed mice using adeno-associated viruses and suggests that TGF-β1 may be a target of Fra2 transcriptional activation. However, it should be noted that there are species differences in the chronic pathological characteristics and lung clearance mechanism between the silicosis model constructed by single high-dose SiO_2_ exposure and human long-term low-dose SiO_2_ occupational exposure, which may affect the clinical relevance. Therefore, we will establish a mouse model of silicosis by long-term low-dose dynamic inhalation, and carry out a prospective cohort study in conjunction with occupational disease prevention and control institutions to analyze the association between peripheral blood Fra2 expression and lung injury.

## CRediT authorship contribution statement

**Yuanmeng Qi:** Writing – review & editing, Writing – original draft. **Jiarui Xia:** Methodology. **Xuesong Zhang:** Data curation. **Xiaoying Li:** Data curation. **Qimeng Li:** Methodology. **Zhenzhen Yang:** Methodology. **Wu Yao:** Supervision. **Changfu Hao:** Data curation, Conceptualization. **Youliang Zhao:** Formal analysis, Data curation, Conceptualization.

## Ethics declaration

The animal experimental protocol used was approved by the Zhengzhou University Animal Research Ethics Committee (ZZUIRB 2021–121).

## Data availability

Data will be made available on request.

## Funding

This work was supported by the 10.13039/501100001809National Natural Science Foundation of China (No. 82173491).

## Conflict of interests

The authors declare no conflict of interests in the study.

## References

[1] Ou L., Zhang P., Huang Z. (2023). Targeting STING-mediated pro-inflammatory and pro-fibrotic effects of alveolar macrophages and fibroblasts blunts silicosis caused by silica particles. J Hazard Mater.

[2] Liu T.T., Sun H.F., Han Y.X., Zhan Y., Jiang J.D. (2024). The role of inflammation in silicosis. Front Pharmacol.

[3] Fan M., Xiao H., Song D. (2022). A novel N-Arylpyridone compound alleviates the inflammatory and fibrotic reaction of silicosis by inhibiting the ASK1-p38 pathway and regulating macrophage polarization. Front Pharmacol.

[4] Ucero A.C., Bakiri L., Roediger B. (2019). Fra-2-expressing macrophages promote lung fibrosis in mice. J Clin Investig.

[5] Roy S., Khanna S., Azad A. (2010). Fra-2 mediates oxygen-sensitive induction of transforming growth factor beta in cardiac fibroblasts. Cardiovasc Res.

