# Supplementary material for: Fra2, a potential therapeutic target for silicosis
Source: Genes Dis. 2025 Jun 18;13(2):101723. doi: 10.1016/j.gendis.2025.101723 (PMC12639824; doi:10.1016/j.gendis.2025.101723)
Supplement: Multimedia component 1 [file mmc1.docx]

**Supplementary Figures**


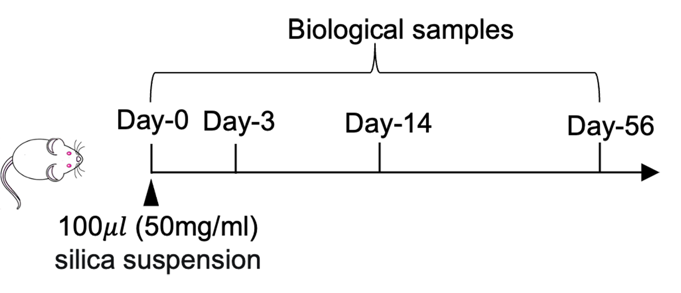


**Fig. S1.** Schematic illustration of the modeling scheme for anthrasilicosis.


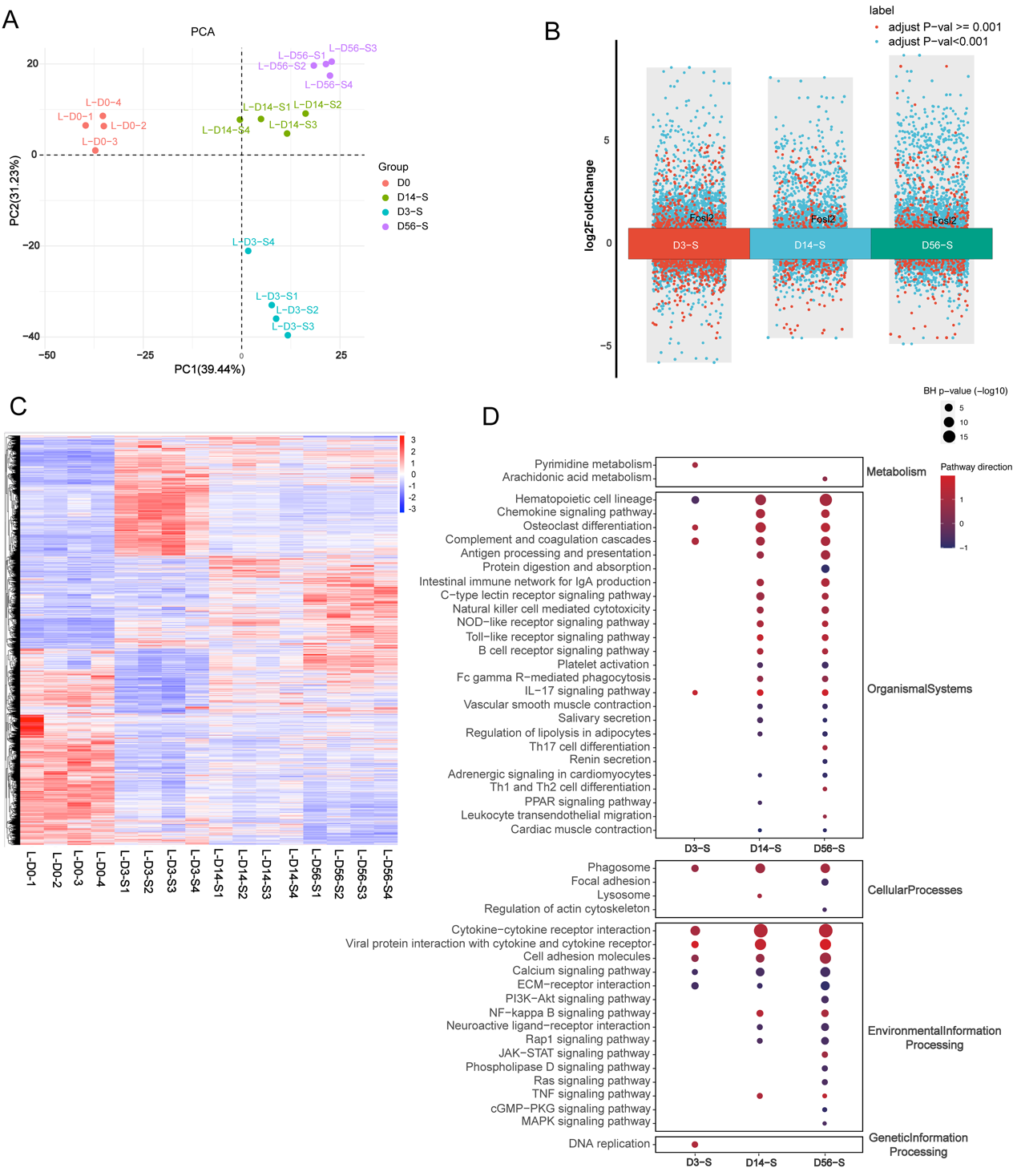


**Fig. S2.** RNA-seq sequencing of mouse lung tissue at various time points. (A) PCA plots of RNA-seq samples (PC1, PC2 represent different components, numbers in parentheses represent the degree of principal component interpretation). (B) Volcano plots showed differential expression profiles of silica-treated and control groups (red dots indicated significantly different up-regulated genes and blue dots indicated significantly different down-regulated genes). (C) Heatmap displayed differential expression profiles for each group. (Red color indicated relatively highly expressed genes and blue color indicated relatively lowly expressed genes). (D) Pathway analysis revealed differential genes in each group. Dot size indicated pathway significance (blue, down-regulated; red, up-regulated).


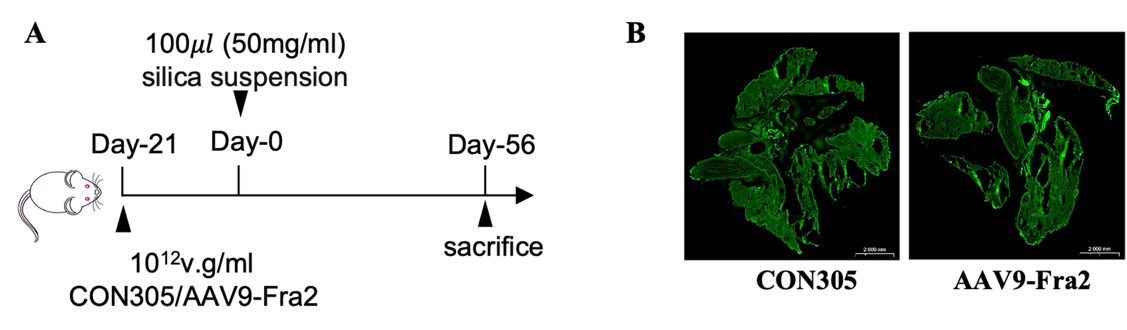


**Fig. S3.** AVV9-Fra2 attenuated silica-induced pulmonary fibrosis. (A) Schematic diagram showed the construction scheme of the AVV9-Fra2 intervention model of silicosis. (B) IF expression was observed in mouse lung tissue after tracheal drip injection of AAV.

**Supplementary Materials and methods**

1. Silicon dioxide dust

SiO_2_ dust (99% purity, particle size 0.5~10 μm), purchased from sigma-Aldrich (Shanghai, China), was sufficiently milled and autoclaved, and then prepared into a SiO_2_ suspension with a final concentration of 50 mg/ml using saline containing penicillin (6000 U/ml).

2. Animal experiment design

Specific pathogen free (SPF) C57/6N mice, weighing 20-25 g, aged 5-6 weeks, were purchased from SPF(Beijing) Biotechnology Co., LTD. All experimental animals were housed in the barrier animal house of Zhengzhou University School of Public Health with 12-hour light and dark cycles. After 1 week of acclimatization in the barrier, 40 mice were randomly divided into a control group (D0) and SiO_2_ groups (D3-S, D14-S, and D56-S) on days 3, 14, and 56 of dust staining. A mouse silicosis model was constructed using the non-exposed tracheal instillation method, with 100μl of SiO_2_ suspension (50 mg/ml) instilleded into the SiO_2_ group and an equal volume of saline instilleded into the control group. The animal experiments complied with the "3R" principle and were approved by the Zhengzhou University Life Sciences Ethics Review Board (ZZUIRB 2021-121).

Forty mice were randomly divided into four groups: CON305+Saline group group, CON305+Silica group, AAV9-Fra2+Saline group and AAV9-Fra2+Silica group. A non-exposed tracheal instillation of AAV9-Fra2（10^12^v.g/ml，100 μl）and an equal dose of empty vector were used. Three mice were randomly executed after three weeks for observation of adeno-associated virus expression in the lungs. After 21 days of AAV9-Fra2 expression, 100 μl of SiO_2_ suspension (50 mg/ml) was titrated in CON305+Silica group and AAV9-Fra2+Silica group, and equal volume of saline was titrated in CON305+Saline group and AAV9-Fra2+Saline group. Biological samples were collected on day 56 after SiO_2_ exposure for subsequent experiments.

3. Euthanization produce

The mice were anesthetized by intraperitoneal injection of sodium pentobarbital (50 mg/kg), and the animals were observed for corneal reflexes, degree of muscle relaxation, and pain response to determine that the required state of anesthesia was achieved for the experiments before proceeding to the subsequent experimental operations.

4. RNA extraction and sequencing

Total RNA was extracted from mouse lung tissue using TRIzol® reagent (Magen). A260/A280 absorbance ratio of RNA samples was measured by Nanodrop ND-2000 (Thermo Scientific, USA). The RIN value of RNA was determined by Agilent Bioanalyzer 4150 (AgilentTechnologies, CA, USA). PE libraries were prepared according to the instructions of ABclonal mRNA-seq Lib Prep Kit (ABclonal, China). The mRNA was purified from 1μg of QC-qualified total RNA using oligo (dT) magnetic beads, and then the mRNA was fragmented in ABclonal First Strand Synthesis Reaction Buffer. Subsequently, the first strand of cDNA was synthesized using mRNA fragments as templates using random primers and reverse transcriptase (RNase H), and then the second strand of cDNA was synthesized using DNA polymerase I, RNAseH, buffer and dNTPs. The synthesized double-stranded cDNA fragments were ligated to the splice sequence and used for PCR amplification. Finally, sequencing was performed using the (NovaSeq 6000) sequencing platform PE150 read length.

5. Bioinformatics analysis

The raw data in fastq format is first processed by a perl script to remove splice sequences, filter out low quality and reads with N ratios greater than 5%, and obtain clean reads that can be used for subsequent analysis. Clean reads were compared with the reference genome using HISAT2 software to obtain mapped reads for subsequent analysis. FeatureCounts is used to calculate the number of reads matched to each gene, and the FPKM value for each gene is calculated based on the length of the gene. Differential expression analysis of genes between groups was performed using DESeq2, and the default screening thresholds for differentially expressed genes were:|log2FC|>0.5 and *P*< 0.05. GO and KEGG enrichment analyses of the differential genes can indicate the functional enrichment of the differential genes. GO function enrichment and KEGG pathway enrichment analyses were performed using the clusterProfiler R software package, and this GO or KEGG function was considered to be significantly enriched when *P*<0.05. For species included in the AnimalTFDB database, the database was filtered based on gene IDs, resulting in transcription factor annotation of differential genes. PPI analysis is used to investigate whether there are interactions between genes, which utilizes the STRING database to obtain interaction relationships between proteins corresponding to genes.

6. Hematoxylin and Eosin staining (HE)

Mouse lung tissues were fixed in 4% paraformaldehyde, paraffin-embedded and cut into 4 μm sections. Sections were dehydrated and immersed in hematoxylin staining solution (Servicebio, China) for 5 min. Sections were then subjected to differentiation in 1% hydrochloric acid alcohol (Servicebio, China) for 2-5s. Eosin staining solution (Servicebio, China) was stained for 2 min. Anhydrous ethanol dehydration for 10 min, air drying, xylene immersion clear for 10 min, air drying. Neutral dendrites were sealed and then images were acquired using an advanced microscope.

7. Masson′trichrome stains (Masson)

Follow the instructions of Masson Trichrome Stain Kit (Solarbio, China). Mouse lung tissues were fixed in 4% paraformaldehyde and paraffin-embedded, and subsequently cut into 4 μm sections. Weigert's iron hematoxylin staining solution was stained for 10 min. Acidic ethanol differentiation solution was differentiated for 15s. The cells were stained with Ponceau fuchsin staining solution for 10min and washed with weak acid working solution for 1min. Wash with aqueous phosphomolybdic acid solution for 2 min and weak acid working solution for 1 min. stain with aniline blue staining solution for 4 min and weak acid working solution for 1 min. Neutral dendrites were sealed and then images were acquired using an advanced microscope.

8. Immunofluorescence (IF)

Paraffin-embedded sections of 4 μm thickness were deparaffinized and placed in EDTA antigen repair solution buffer (Proteintech, China) for antigen repair. 3% BSA (Solarbio, China) evenly covered the tissues and closed for 30 min at room temperature. The corresponding primary antibody dilutions anti-Fra2 antibody (1:100, Affinity, China), anti-F4/80 antibody (1:50, Proteintech, China) and Anti- TGF-β1 antibody (1:100, Proteintech, China) were added dropfold. China), or anti-α-SMA antibody (1:300, Servicebio, China), sections were laid flat ina wet box and incubated overnight. The secondary antibody (ThermoFisher, USA) of the corresponding species was added dropwise and incubated for 1h at room temperature away from light. Appropriate amount of DAPI staining solution (Servicebio, China) was added dropwise, and incubated at room temperature and away from light for 10 min. After sealing the slices with anti-fluorescence quencher, the slices were observed by fluorescence microscope and the images were collected.

9. Immunohistochemistry (IHC)

4 μm paraffin-embedded sections were deparaffinized and placed in sodium citrate buffer (Solarbio, China) for antigen repair. 3% BSA (Solarbio, China) was used for 30 min at room temperature for containment. Add the corresponding primary antibody dilution anti-Fra2 Antibody (1:200, Affinity, China), and incubate the sections at 4°C overnight in a wet box. The sections were incubated overnight at 4°C in a wet box with a drop of DBA color development solution (ThermoFisher, USA), and left to develop the color for 2 min. Hematoxylin (Solarbio, China) was re-stained for 3 min and dehydrated. Neutral gum was used to seal the slices and then images were acquired using an advanced microscope.

10. Immunoblotting assay (Western blot)

Radioimmunoprecipitation assay buffer (RIPA; Boster Biological Technology, China) containing 1× Phenylmethanesulfonyl fluoride (PMSF; Boster Biological Technology, China) lysate was used to extract the total protein from lung tissues of whole mice. The protein concentration in the lysate was determined using the BCA protein assay Kit (Boster Biological Technology, China). Proteins were separated by polyacrylamide gel electrophoresis (SDS-PAGE) and transferred to PVDF membrane (Merck, Germany). Specific primary antibodies were used, including: anti-Fra2 antibody (1:1000, Affinity, China), anti-TGF-β1 antibody (1:1000, Proteintech, China), anti-α-SMA antibody (1:2000, Servicebio, China) China), anti-Collagen Ι antibody (1:1000, Abcam, UK), anti-CTGF antibody (1:1000, BOSTER, China) and anti-GAPDH antibody (1:1000, Proteintech, UK). China) were incubated overnight at 4°C. Subsequent incubation with the corresponding secondary antibody (1:10000, Proteintech, China) was performed at room temperature for 1 h. Chemiluminescence signals were detected by a chemiluminescence instrument (e-BLOT Touch Imager, China).

11. Quantitative reverse-transcription polymerase chain reaction (RT-qPCR)

Total RNA was extracted using RNAiso Plus (TaKaRa, Japan) reagent. RNA was reverse transcribed to generate cDNA using the reverse transcription kit PrimeScript RT reagent Kit (TaKaRa, Japan). RT-qPCR was performed using the quantification kit TB Green Premix ExIIKit (TaKaRa, Japan). Changes in the expression levels of related genes were calculated using the 2^-ΔΔCT^ method after normalization to glyceraldehyde-phosphate dehydrogenase (GAPDH). All gene primer sequences are shown in Table 1.

Table1 Primer sequence table

| Primer | Sequence | Prodsize(bp) |
| --- | --- | --- |
| GAPDH | Reverse: 5′- AGGTCGGTGTGAACGGATTTG -3′ | 123 |
|  | Reverse:5′-TGTAGACCATGTAGTTGAGGTCA -3′ |  |
| FN1 | Forward: 5′ - ATGTGGACCCCTCCTGATAGT-3′ | 124 |
|  | Reverse: 5′ - GCCCAGTGATTTCAGCAAAGG-3′ |  |
| COL1A1 | Forward:5′-TGAACGTGGTGTACAAGGTC-3′ | 243 |
|  | Reverse:5′-CCATCTTTACCAGGAGAACCAT-3′ |  |
| CTGF | Forward:5’ - CAGCGGTGAGTCCTTCCAAA - 3’  Reverse: 5’ - GGCTCGCATCATAGTTGGGT- 3’ | 259 |
| ACTA2 | Forward:5’ - GGCACCACTGAACCCTAAGG - 3’  Reverse: 5’ - ACAATACCAGTTGTACGTCCAGA - 3’ | 135 |
| Fra2 | Forward:5′- GGAGACCGAGGAGCTGGAAGAG -3′ | 108 |
|  | Reverse: 5′- CCGTGAGCCACCAACATGAACTC -3′ |  |
| TGF-β1 | Forward:5′- ACCGCAACAACGCCATCTATGAG -3′ | 105 |
|  | Reverse: 5′- GGCACTGCTTCCCGAATGTCTG -3′ |  |

12. HYP content in mouse lung tissue

Follow the instructions provided by the manufacturer for the Hydroxyproline Content Assay Kit (Solarbio, China). Weighed 0.2 g of lung tissue samples that had been sheared were boiled in 2 ml of the extraction solution for 2 to 6 h until there were no visible large clumps. After cooling, adjust the pH value with NaOH to 6~8, and then dilute with distilled water to 4 ml. Finally, centrifuge at 16000rpm, 25°C for 20 min, and take the supernatant to determine the OD value.

13. Statistical analysis

Statistical analyses were performed using SPSS 21.0 software. Comparisons between samples of two groups were made using Student's t-test, and comparisons between multiple groups were made using one-way ANOVA. All data are expressed as mean ± standard deviation. Test level α=0.05, *P*<0.05 was considered statistically significant.
